# List of Accidents Admitted into the Pennsylvania Hospital, from March 7th to March 21st, 1838

**Published:** 1838-03-28

**Authors:** 


*List of accidents admitted into the Pennsylvania Hospital, from March 7th to March* 21*st,* 1838.

One incised wound of the lip, opening the coronary artery: a single suture was applied, passing through the artery, and the wound healed in three days. One lacerated wound of the scalp. One wound of the eye, from a stone thrown by a blast of gunpowder; the upper portion of the iris was torn from its adhesions; severe inflammation followed; V. S. twice, to fainting, purging, a blister, and rigid diet—doing well. One contusion of the side from a fall, discharged, cured in seven days. One sprained ankle. One fractured clavicle, seven days after the injury, discharged in seven days, union perfectly formed. One bite of a dog in the hand: the part involved was cut out. One sprain of the wrist joint. One lacerated wound of the hand, with fracture of the fingers.

